# Transcriptome dynamics in early in vivo developing and in vitro produced porcine embryos

**DOI:** 10.1186/s12864-021-07430-7

**Published:** 2021-02-27

**Authors:** Vera A. van der Weijden, Meret Schmidhauser, Mayuko Kurome, Johannes Knubben, Veronika L. Flöter, Eckhard Wolf, Susanne E. Ulbrich

**Affiliations:** 1 ETH Zurich, Animal Physiology, Institute of Agricultural Sciences, Universitätstrasse 2, CH-8092 Zurich, Switzerland; 2 Chair for Molecular Animal Breeding and Biotechnology, and Center for Innovative Medical Models (CiMM), LMU Munich, Munich, Germany; 3 Physiology Weihenstephan, Technical University Munich, Freising, Germany

**Keywords:** Transcriptomics, Porcine, Embryo development, In vivo embryo development, in vitro fertilization

## Abstract

**Background:**

The transcriptional changes around the time of embryonic genome activation in pre-implantation embryos indicate that this process is highly dynamic. In vitro produced porcine blastocysts are known to be less competent than in vivo developed blastocysts. To understand the conditions that compromise developmental competence of in vitro embryos, it is crucial to evaluate the transcriptional profile of porcine embryos during pre-implantation stages. In this study, we investigated the transcriptome dynamics in in vivo developed and in vitro produced 4-cell embryos, morulae and hatched blastocysts.

**Results:**

In vivo developed and in vitro produced embryos displayed largely similar transcriptome profiles during development. Enriched canonical pathways from the 4-cell to the morula transition that were shared between in vivo developed and in vitro produced embryos included oxidative phosphorylation and EIF2 signaling. The shared canonical pathways from the morula to the hatched blastocyst transition were 14–3-3-mediated signaling, xenobiotic metabolism general signaling pathway, and NRF2-mediated oxidative stress response. The in vivo developed and in vitro produced hatched blastocysts further were compared to identify molecular signaling pathways indicative of lower developmental competence of in vitro produced hatched blastocysts. A higher metabolic rate and expression of the arginine transporter *SLC7A1* were found in in vitro produced hatched blastocysts.

**Conclusions:**

Our findings suggest that embryos with compromised developmental potential are arrested at an early stage of development, while embryos developing to the hatched blastocyst stage display largely similar transcriptome profiles, irrespective of the embryo source. The hatched blastocysts derived from the in vitro fertilization-pipeline showed an enrichment in molecular signaling pathways associated with lower developmental competence, compared to the in vivo developed embryos.

**Supplementary Information:**

The online version contains supplementary material available at 10.1186/s12864-021-07430-7.

## Background

In pigs and humans, embryo development is under maternal control until the 4-cell stage [1, 2]. Until this stage, proteins and RNA, stored in the oocyte, control embryo development. The embryonic cells contain inactive nucleolus precursor bodies [3]. After embryonic genome activation (EGA), embryonic control commences at around day 3 post fertilization [1]. The inactive nucleolus precursor bodies transform into functional nucleoli [3]. These nucleoli exhibit functional components including fibrillar centers containing rRNA genes and enzymes facilitating transcription, dense fibrillary components containing nascent rRNA and enzymes required for its processing, and granular components containing large ribosomal subunits and enzymes required for packaging [3]. Compaction is initiated in the oviduct by the 8- to 16-cell stage, and by day 4, the morula is formed [1, 3]. Blastulation takes place in the uterus and during this process, the outer embryonic cells connect by tight junctions and desmosomes, thereby sealing the expanding blastocoel [3]. The blastocyst is formed by day 5 after fertilization and consists of lipid containing inner cell mass and trophectoderm cells [1, 3]. At day 7 of development, the embryo hatches from the zona pellucida and increases in size until day 10 of development [4]. Up to the blastocyst stage, embryos can be produced and cultured in vitro. Despite ongoing efforts to improve the quality of in vitro produced blastocysts, these embryos are less competent than in vivo developed blastocysts [5]. Therefore, it is important to understand which molecular pathways are affected by the in vitro embryo production pipelines. In vivo*,* the embryo starts to rapidly elongate by day 11 of development and secretes estradiol-17β (E2) as primary recognition of pregnancy signal [6]. The secretion of embryonic E2 coincides with the endometrial expression of E2-regulated genes [7]. The transition of the hatched blastocyst to an elongated embryo takes place rapidly [8].

A dynamic and embryonic developmental stage-specific mRNA expression has been shown in various species [9, 10]. Single-cell RNA sequencing of murine and bovine embryos revealed a transcriptional variation of single blastomeres [10, 11]. Single murine blastomeres showed an increasing transcriptional variation with developmental progression [10]. Similar findings have been reported for stem cell differentiation. Stem cells had a more uniform transcriptome profile compared to differentiated cells [12]. The single cell reconstruction of murine preimplantation development showed distinct developmental stage-dependent clusters, i.e., 2-cell, 4-cell, 8-cell and 16-cell stage embryos, while single cells from the early, mid and late blastocyst clustered together [10]. In pigs, the transcriptional changes of embryos around the time of EGA (2- and 4-cell stage embryos) have been investigated in both in vivo developed and in vitro produced whole embryos, aiming at gaining insights into the mechanisms that lead to reduced developmental potential of in vitro produced embryos [13]. In vitro produced embryos displayed altered transcript levels for apoptotic factors, cell cycle regulation factors and spindle components, as well as transcription factors, collectively contributing to reduced developmental competence of in vitro produced embryos [13]. To understand the species-specific regulatory networks involved in EGA, the first lineage commitment and the primitive endoderm differentiation, Cao et al. (2014) evaluated the expression of putative inner cell mass (ICM) and trophectoderm (TE) markers in oocytes, 1-cell, 2-cell, 4-cell, 8-cell embryos, morulae, early blastocysts, and expanded blastocysts [14]. By comparing the transcriptome changes with those of mouse and human pre-implantation embryos, a unique pattern was found in pig embryos [14]. In addition, the global gene expression pattern was different in somatic cell nuclear transfer (SCNT) embryos compared to in vivo developed embryos [14]. The pig EGA was confirmed to take place at the 4-cell stage, while this only appeared at the 8-cell stage in SCNT embryos [14]. The differentially expressed genes from the hatched blastocyst to tubular and filamentous embryos included glycolytic enzymes that are potentially regulated by estrogen [15, 16].

To date, the developmental competence, as well as pregnancy rates after transferring in vitro produced porcine embryos remain low [17]. This can, in part, be attributed to aberrant chromatin dynamics [18]. Compared to in vivo produced embryos, in vitro produced embryos showed developmental stage-dependent altered chromatin dynamics. Already at the two-cell stage, they displayed aberrant chromatin-nuclear envelope interactions [18]. In vitro produced embryos showed global chromatin remodeling imperfections and failed to establish a proper first lineage segregation at the blastocyst stage [18]. To improve the developmental competence of in vitro embryos, it is crucial to elucidate their transcriptional profile during pre-implantation development. In this study, we aimed at furthering the understanding of early embryo development, and to identify molecular pathways that could explain lower developmental competence of in vitro produced hatched blastocysts.

## Results

### Samples and RNA sequencing

**Fig. 1:**
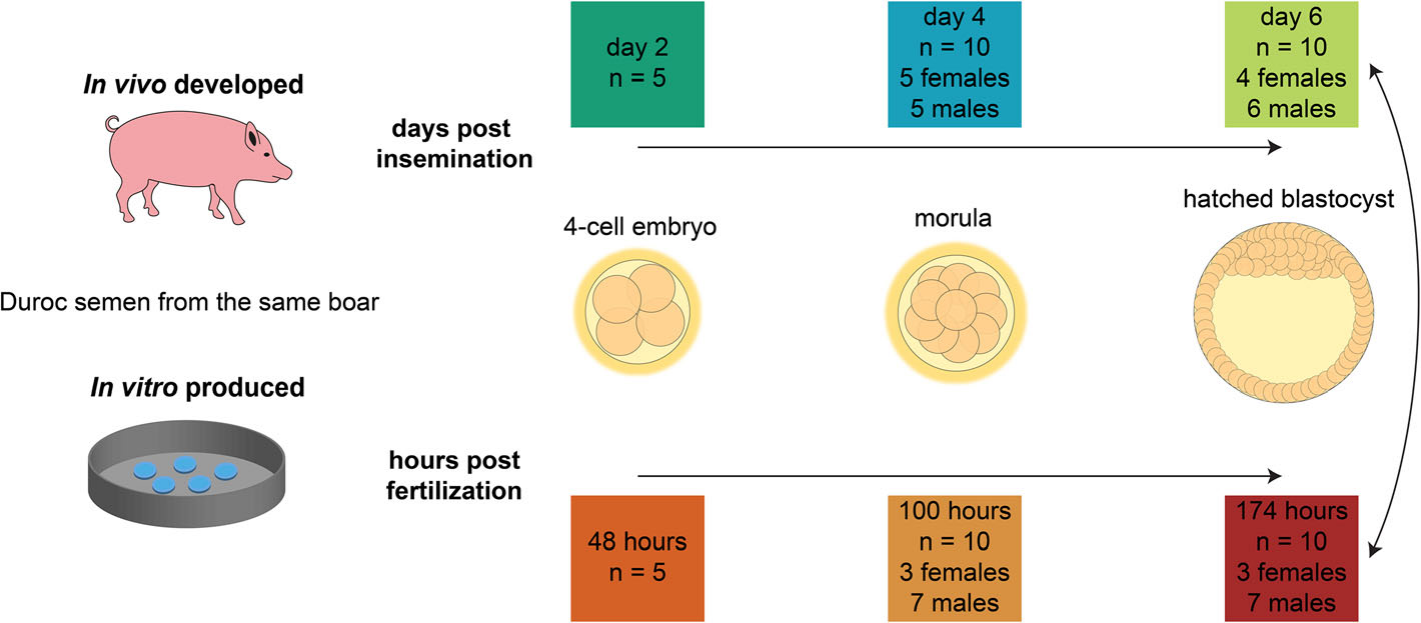
Experimental set-up for single embryo RNA-sequencing. The arrows indicate the between-group analyses

RNA sequencing was performed using 50 single embryos (Fig. 1).

A total of 1405 million raw reads was obtained after RNA sequencing, with a duplication rate of 63 ± 7% (mean ± SD) and a GC content of 45 ± 1% (mean ± SD). The mapping rate after quality filtering was 84 ± 6% (mean ± SD). The number of detected transcripts, defined as any transcript with at CPM > 0.1, increased with developmental progression for the in vivo produced embryos, while it decreased for the in vitro produced embryos (Additional file 1). The low number of detected transcripts for the 4-cell in vivo embryos might be the consequence of analyzing 4-cell embryos with a reduced RNA quality, relatively low input and cDNA yield during library preparation (Additional file 2). Given the differences in RNA quality as assessed by the cDNA profile, library smear analyses, and read alignment at the 4-cell, as well as at the morula stage (Additional file 2 and 3), the in vivo developed and in vitro produced embryos were analyzed separately and were not compared to each other. To identify in vitro fertilization pipeline-induced transcriptome differences, the hatched blastocysts were used for an in vivo developed versus in vitro produced comparison.

### Developmental transcriptome dynamics

**Fig. 2:**
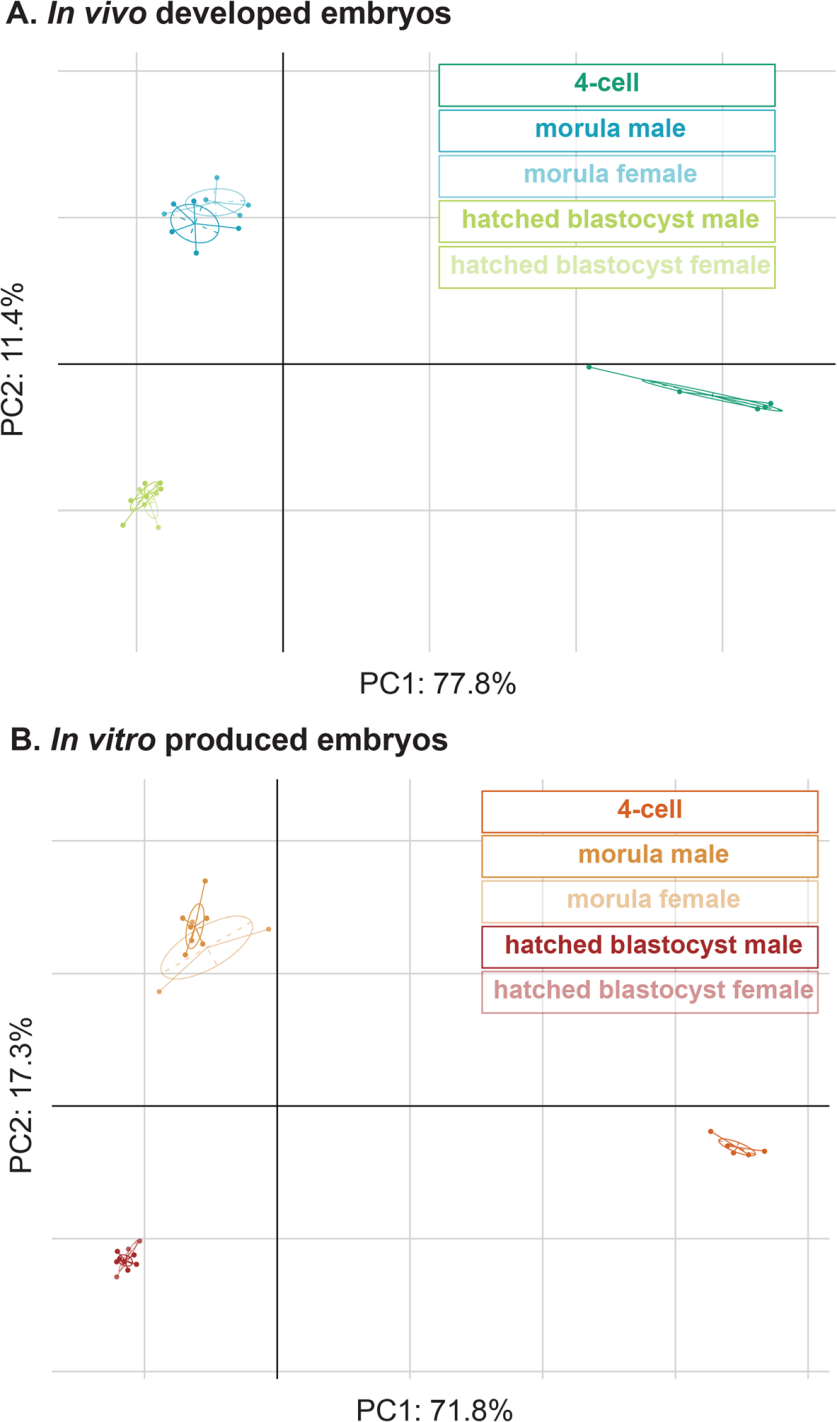
Between-group analyses of the 4-cell stage embryos, morulae and hatched blastocysts of **a**. In vivo developed embryos, and **b**. In vitro produced embryos

To provide a developmental stage-specific overview, global developmental transcriptome dynamics were investigated. Principal component analyses (PCA) were performed separately for the in vivo developed and in vitro produced embryos and showed a clear developmental stage-specific clustering of the embryos (Fig. 2a and b). For the in vivo developed embryos, PC1 and PC2 explained 77.8 and 11.4% of the variance in transcript levels. For the in vitro produced embryos, PC1 and PC2 explained 71.8 and 17.3% of the variance. The in vivo 4-cell embryos displayed a larger degree of transcriptional heterogeneity than the in vitro 4-cell embryos. The morulae and hatched blastocysts were sexed based on the expression of Y-chromosome specific transcripts. At the morula stage, male and female embryos clustered together, yet the clusters were not fully overlapping. At the blastocyst stages, the male and female clusters were fully overlapping.

### In vivo and in vitro embryonic developmental dynamics

**Fig. 3:**
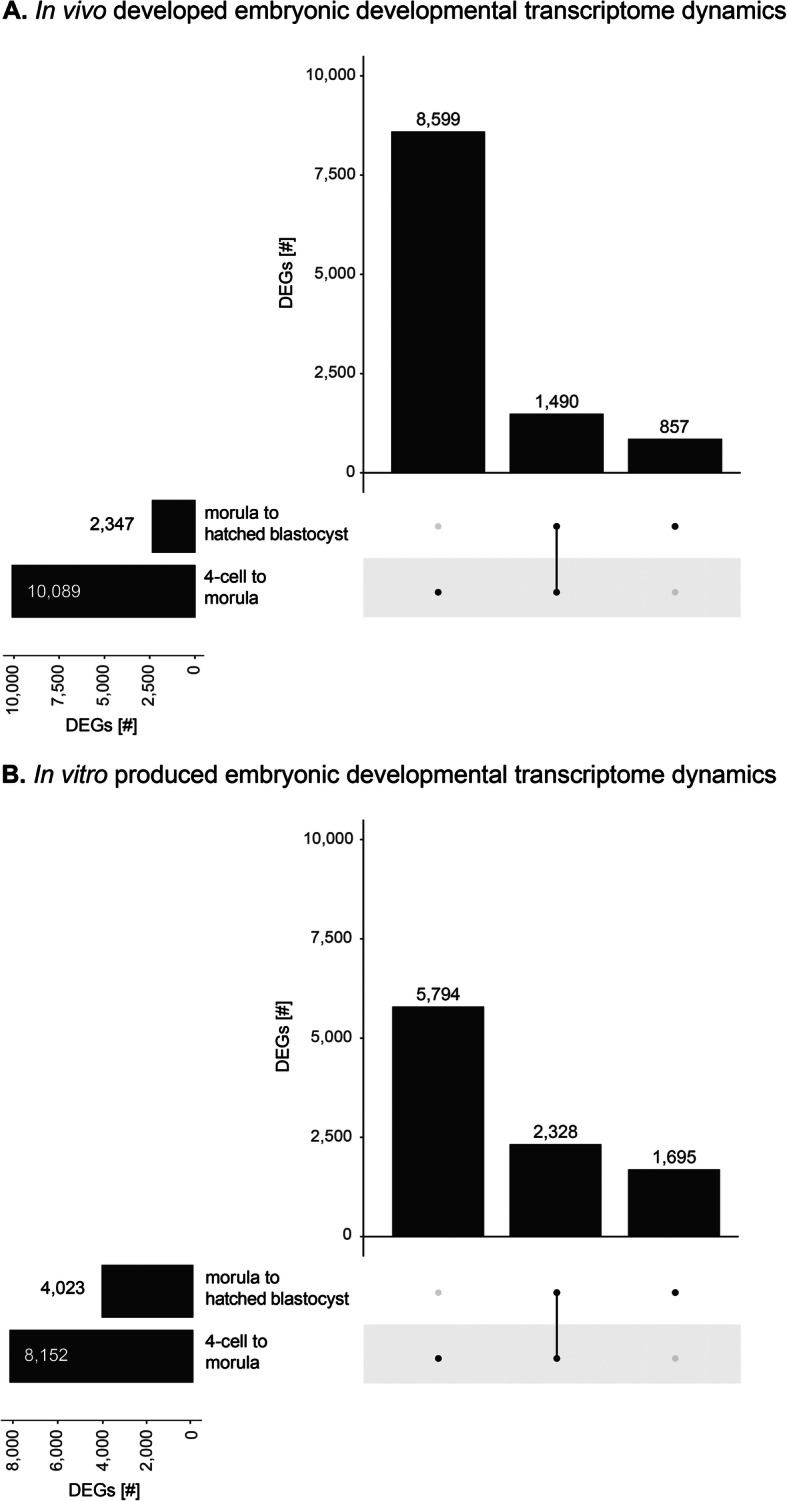
Upset plot displaying the differentially expressed genes during embryo development from the 4-cell to the morula stage and the morula to the hatched blastocyst stage in **a**. In vivo developed embryos, and **b**. In vitro produced embryos

The developmental transcriptome dynamics were further analyzed by identifying differentially expressed genes (DEGs) between the 4-cell and morula stage, and the morula and hatched blastocyst stage for both the in vivo developed and in vitro produced embryos. The number of DEGs was higher between the 4-cell to morula stage, than for the morula to hatched blastocyst stage (Fig. 3). For the in vivo embryos, 10089 and 2347 DEGs were identified between the 4-cell to the morula stage and the morula stage to the hatched blastocyst stage, respectively (Fig. 3a). For the in vitro embryos, 8152 and 4023 DEGs were identified between the 4-cell to the morula stage and the morula stage to the hatched blastocyst stage, respectively (Fig. 3b).

**Fig. 4:**
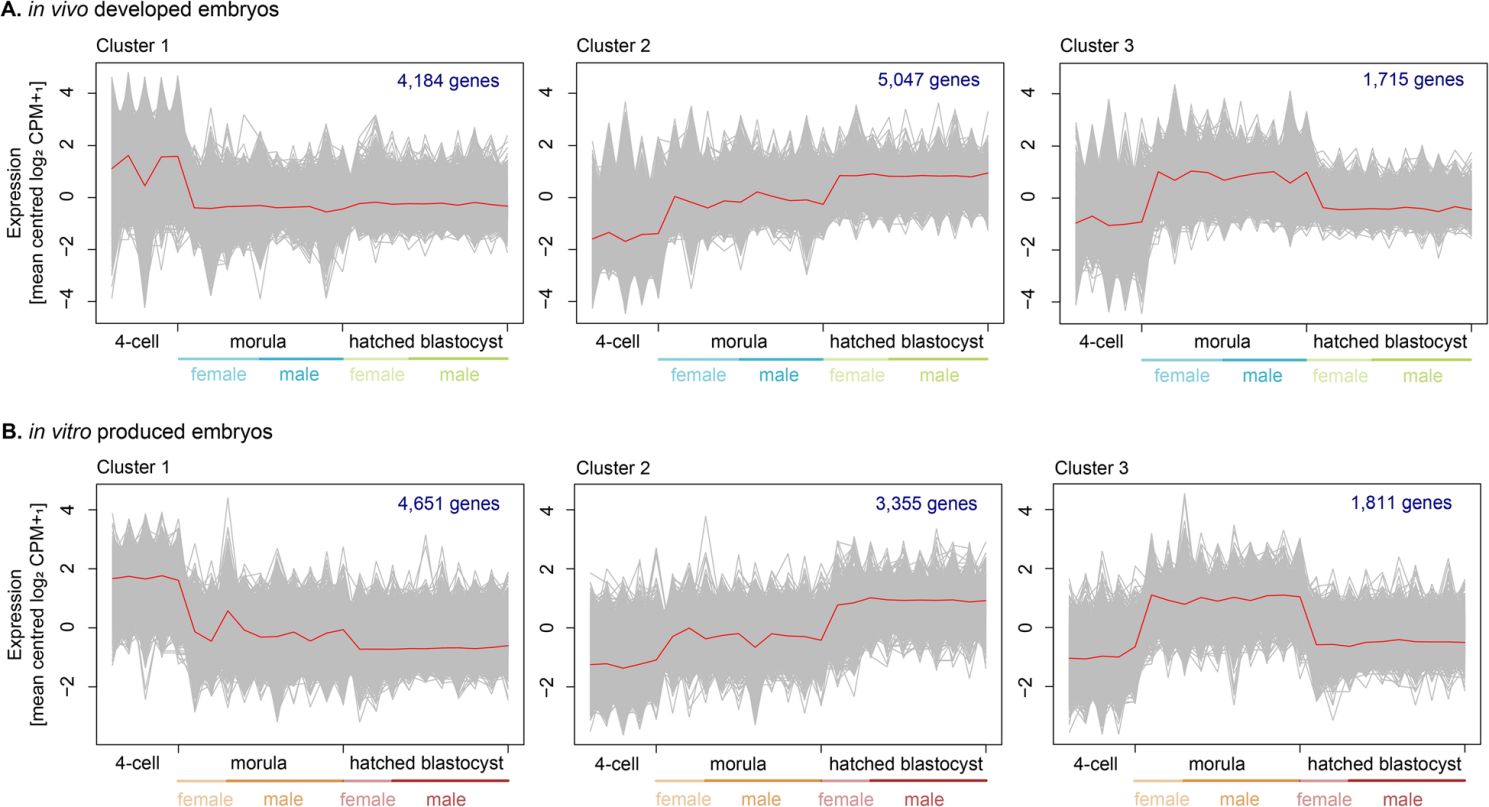
Transcriptome dynamics during development displayed by a self-organizing tree algorithm for **a.** In vivo developed embryos, and **b.** In vitro produced embryos. The number of genes per cluster and the embryonic sex of the morulae and hatched blastocysts are indicated

The developmental dynamics were assessed with a self-organizing tree algorithm (Fig. 4a and b). For both the in vivo and in vitro produced embryos, the detected transcript expression changed from the 4-cell to the morula stage. The transcripts in cluster 1 decreased from the 4-cell to the morula stage, and remained low at the hatched blastocyst stage. The transcripts in cluster 2 displayed a gradual increase with developmental progression. The transcripts in cluster 3 were increased at the morula stage, while remaining low at the 4-cell and the hatched blastocyst stage.

### Biological functions of embryonic developmental dynamics

**Fig. 5:**
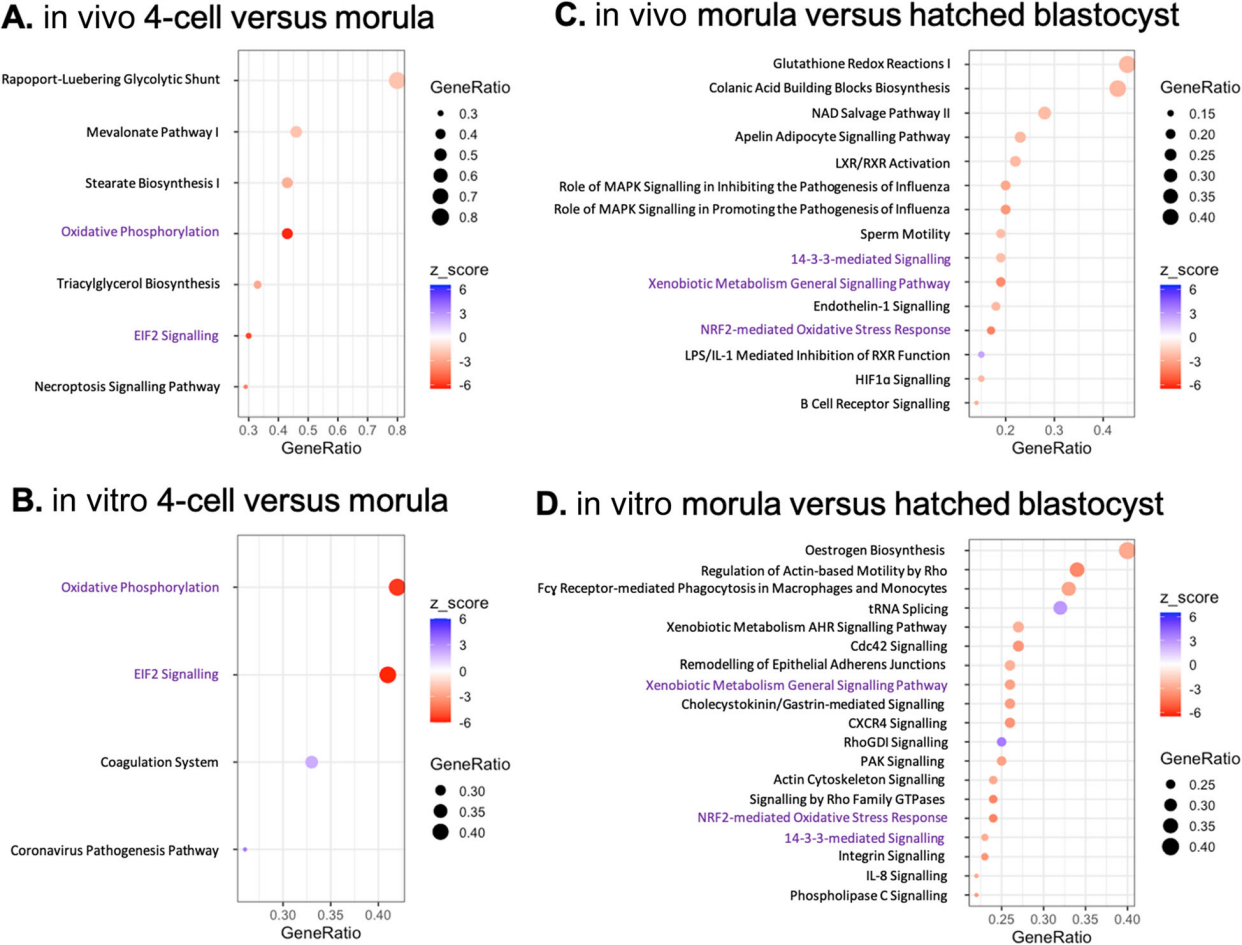
Enriched canonical pathways. Red (−) dots represent canonical pathways of which genes were significantly lower expressed in the 4-cell versus morula and morula versus hatched blastocysts, and blue (+) represent canonical pathways of which genes were significantly higher expressed in the 4-cell versus morula or morula versus hatched blastocysts. The GeneRatio indicates the proportion of DEGs that were identified in an enriched canonical pathway. Shared enriched canonical pathways in both in vivo developed and in vitro produced embryos at the 4-cell versus morula or morula versus hatched blastocyst stage are indicated in purple

To gain insight into the biological functions of the DEGs, a canonical pathway enrichment analysis was conducted (Fig. 5). In both the in vivo and the in vitro produced 4-cell to morula stage embryos, there was a significant enrichment of oxidative phosphorylation and EIF2 signaling. From the morula to the hatched blastocyst stage, the DEGs in the pathways 14–3-3-mediated signaling, xenobiotic metabolism general signaling pathways, and NRF2-mediated oxidative stress response were all higher expressed at the hatched blastocyst stage for both the in vivo and in vitro produced embryos.

### In vivo and in vitro differences at the hatched blastocyst stage

The in vivo and in vitro hatched blastocysts were compared, as the embryos displayed similar cDNA profiles, library smears and alignment coverages for the most abundant transcripts at this developmental stage (Additional file 2 and 3). Embryos at this stage of development are thought to be more alike than at earlier stages, as time differences related to fertilization at earlier stages contribute more substantially to the actual developmental stage.

**Fig. 6:**
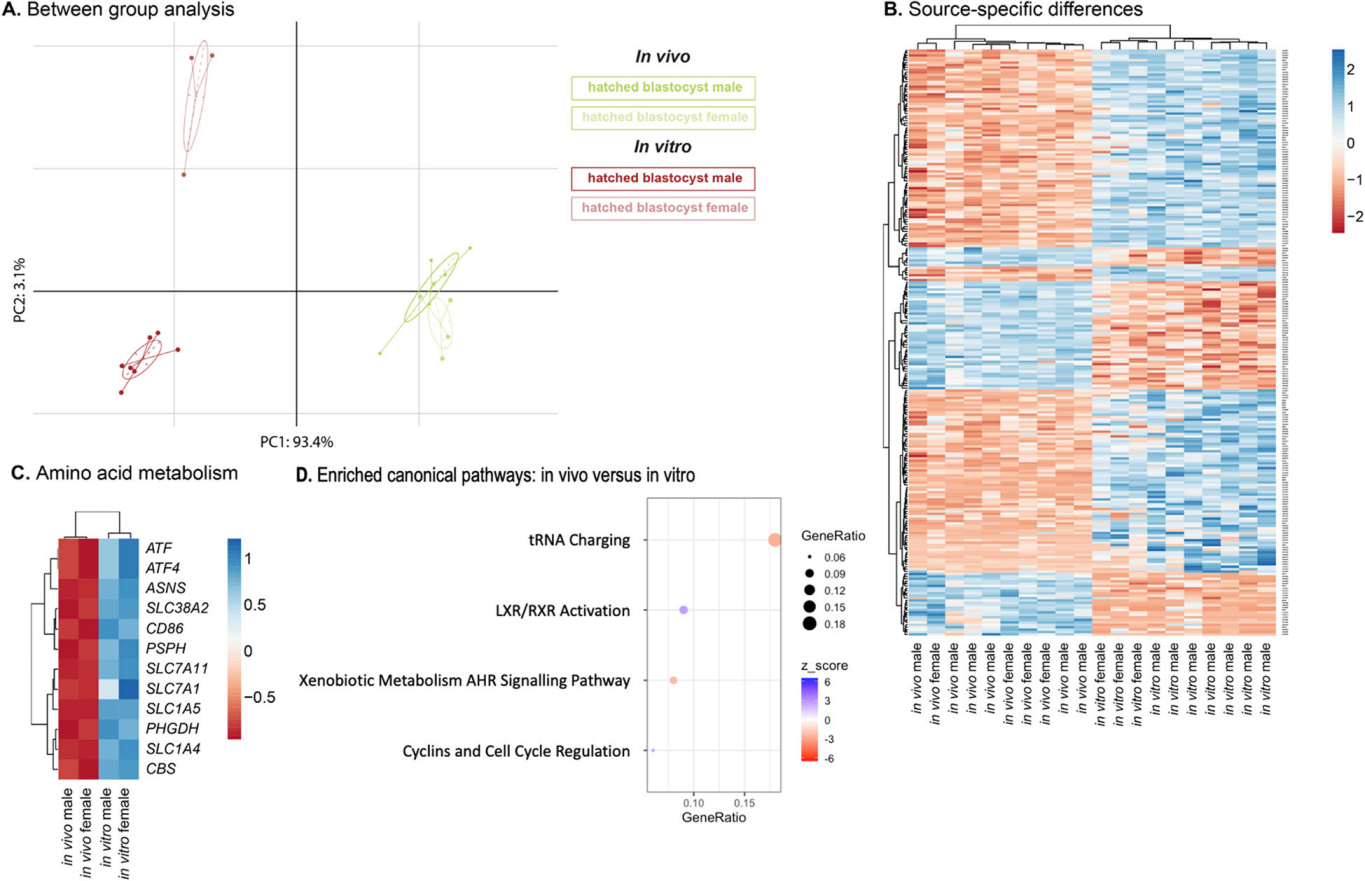
**a.** Between group analysis of in vivo and in vitro female and male hatched blastocysts. **b.** Heatmap displaying the 243 DEGs observed by comparing in vivo produced versus in vitro developed females and the in vivo produced versus in vitro developed males. **c.** Heatmap displaying genes involved in amino acid metabolism, and **d.** Significantly enriched canonical pathways between the in vivo developed and in vitro produced hatched blastocysts. Red (−) dots represent canonical pathways of which genes were significantly less expressed in the in vivo embryos, while blue (+) represent canonical pathways of which genes were significantly higher expressed in the in vivo embryos. The GeneRatio indicates the proportion of DEGs that were identified in an enriched canonical pathway

At the hatched blastocyst stage, the selection of developmentally competent embryos has already taken place. Yet, we unraveled in vitro fertilization pipeline-induced sex-specific differences. The in vivo developed female and male hatched blastocysts clustered largely together (Fig. 6a). They were separated from the in vitro hatched blastocyst in a sex-specific manner by principal component 1. While 33 DEGs were identified between the female in vivo and in vitro produced embryos, 241 DEGs were identified between the male in vivo and in vitro produced embryos. Figure 6b displays the difference between in vivo developed and in vitro produced embryos in a sex-independent manner. There were no DEGs when comparing male and female embryos for either in vivo developed or in vitro produced embryos. By comparing the female in vivo developed versus in vitro produced embryos, the DEGs inositol polyphosphate multikinase (*IPMK*) and Rac family small GTPase 1 (*RAC1*) were specific to this comparison. The other 31 DEGs were also discovered by comparing the in vivo and in vitro male hatched blastocysts. These genes were involved in amino acids transport, synthesis and metabolism, and similarly expressed in both female and male embryos (Fig. 6c). Both male and female in vivo derived embryos had a lower expression of genes involved in amino acid transport, synthesis and metabolism compared to the male and female in vitro produced embryos.

When disregarding the sex of the embryos and emphasizing on the embryo source, a total of 398 DEGs were identified. The persistent difference between in vivo developed and in vitro produced embryos at the hatched blastocyst stage were illustrated by an enrichment of four canonical pathways (Fig. 6d). Except for a higher expression in in vivo versus in vitro hatched blastocysts of DEGs involved in cyclins and cell cycle regulation and LXR/RXR activation, the DEGs involved in tRNA charging and xenobiotic metabolism AHR signaling pathways were higher expressed in in vitro than in in vivo hatched blastocysts.

## Discussion

### Transcriptome dynamics during early embryo development

Early developing porcine embryos displayed a great adaptive capacity towards their environment, evidenced by largely similar transcriptome dynamics observed in both in vivo developed and in vitro produced embryos. in vitro produced embryos offer the opportunity to study molecular pathways of interest in a developmental-stage specific manner, as there is a higher degree of certainty regarding the time of fertilization compared to in vivo developed embryos. However, developmental rates and embryo competence of in vitro produced embryos are still lower compared to their in vivo developed counterparts [5]. A number of factors are known to contribute to embryo development. The presence of cumulus cells during maturation facilitates full oocyte maturation [19]. In pigs, the presence of cumulus cells during oocyte maturation is essential for oocyte maturation, fertilization and subsequent embryo development [20]. The discrepancy in embryo development between in vivo developed and in vitro produced embryos at early post-fertilization developmental stages might be explained by the use of a pool of non-selected oocytes of overall lower competence for in vitro maturation, compared to those selected for ovulation, and the effects of in vitro maturation on oocyte quality. A higher blastocyst rate has previously been shown after oocyte maturation under a 20% oxygen atmosphere [21]. However, blastocyst quality assessed by the expression of genes related to metabolism (*GLUT1* and *LDHA*), antioxidant response (*SOD2* and *GPX1*), growth factors and apoptosis (*IGF2R*, *BCL2* and *BAX*), methylation (*DNMT3B*), and blastocyst quality (*AKR1B1*, *POU5F1* and *CDX2*) were not affected [21]. In addition, the blastocyst rates of in vivo and in vitro matured rabbit oocytes did not significantly differ, while at earlier developmental stages, the in vivo embryo development rates were significantly higher than observed for embryos produced with in vitro matured oocytes [22]. Thus, while oocyte quality and competence, and subsequent embryo development are affected by the maturation conditions, only minor transcriptional differences have been reported at the hatched blastocyst stage [23]. In line with previous findings, we found more similar transcriptome profiles at later developmental stages. At the hatched blastocyst stage, only limited transcriptional differences persisted. Additionally, the developmental-stage specific differences were more pronounced than the sex-specific differences, as previously described by Zeng et al. (2019), studying the transcriptome dynamics in in vivo developed day 8, 10, and 12 porcine embryos [16].

### Early porcine embryo development

The early embryo development was studied at the 4-cell, morula and hatched blastocyst stage for both in vivo developed and in vitro produced embryos. Previously, porcine embryos after EGA have been shown to display an increased abundance of transcripts involved in, among others, transcription [13]. Both the in vivo developed and in vitro produced 4-cell to morula transition was characterized by an enrichment of oxidative phosphorylation and EIF2 signaling. An increase in oxidative phosphorylation with developmental progression has previously been reported for mouse embryos [24]. Oxidative phosphorylation accounted for 60–70% of consumed oxygen in blastocysts, compared to 30% of consumed oxygen in cleavage stage embryos [24]. In addition, oxygen consumption of in vivo bovine blastocysts increased with increasing morphological quality and developmental stage [25]. Yet, in vitro produced embryos displayed a higher oxygen consumption, which was related to lower pregnancy rates [25]. Thus, in vitro morulae seem developmentally competent, as they display increased transcription of genes related to oxidative phosphorylation, as observed for the in vivo embryos. EIF2 signaling has previously been shown to be downregulated in parthenogenetically activated expanded porcine blastocysts compared to in vivo developed embryos, evidencing a correlation between aberrant EIF2 signaling and reduced developmental competence [26]. EIF2 signaling was upregulated in morulae compared to 4-cell embryos, irrespective of embryo source, evidencing cell growth and proliferation [27].

During the morula to the hatched blastocyst transition, both in vivo developed and in vitro produced embryos displayed an enrichment of the pathways 14–3-3-mediated signaling, xenobiotic metabolism general signaling pathways, and NRF2-mediated oxidative stress response. The 14–3-3 signaling plays a role in normal growth and development [28], cell polarity [29], and cell fate [30]. In bovine, the NRF-2 mediated oxidative stress response is enriched in competent blastocysts [31], and the functions and processes related to the NRF-2 mediated oxidative stress response and oxidative phosphorylation pathways have been suggested to be related to developmental competence [32]. The enrichment of the shared signaling pathways in both in vivo developed and in vitro produced embryos during development from the morula to hatched blastocyst stage appeared to be indicative of largely similar developmental transcriptional profiles, potentially related to embryo competence.

### In vivo developed versus in vitro produced hatched blastocysts

The differences between in vivo developed and in vitro produced hatched blastocysts were investigated to understand persisting transcriptional differences and their relationship to embryo competence. Whitworth et al. (2005) previously reported DEGs in porcine blastocyst stage embryos by comparing in vivo developed and in vitro produced embryos [23]. Unlike the difference in expression of *HMGB1* they reported, we did not find a difference in its expression between in vivo developed and in vitro produced hatched blastocysts. The expression of *HMGB1* has been associated with the number of nuclei per embryo [23], suggesting that the stage of our hatched blastocysts is likely similar, thereby allowing the comparison between in vivo developed and in vitro produced embryos at this developmental stage. Likewise, there was no significant difference in the expression of *ATP5A1* between in vivo developed and in vitro produced hatched blastocysts. The expression of *ATP5A1* has previously been used to indicate differences in metabolic rates in in vivo developed and in vitro produced blastocysts [23]. In addition, 71% of genes related to cellular metabolism were reported to be upregulated in in vivo developed compared to in vitro produced porcine blastocysts [33]. The in vitro hatched blastocysts in this study displayed a significant increase in amino acid metabolism. Among the genes related to amino acid metabolism, the arginine transporter *SLC7A1* has previously been reported to be significantly upregulated in in vitro produced embryos compared to in vivo developed embryos [34]. Porcine embryos deplete arginine from the culture medium at a higher rate at the expanded blastocyst stage compared to early blastocysts [35]. The arginine concentration in the embryo culture medium used in this study was at 0.1 mM [36]. It has previously been shown that adding arginine to a final concentration of 0.36 mM to the embryo culture medium decreased the *SLC7A1* transcript level in in vitro produced embryos to a level comparable to the in vivo developed embryos [34]. In our study, the in vitro produced hatched blastocyst displayed a higher transcript expression of genes related to tRNA charging and xenobiotic metabolism AHR signaling pathways. The in vivo developed embryos displayed a higher transcript expression of genes related to cyclins and cell cycle regulation, and LXR/RXR activation. in vitro produced porcine blastocyst have previously been reported to display a higher transcript expression of genes involved in, among others, mRNA transcription, nucleotide metabolism, DNA metabolism, amino acid metabolism, and lipid metabolism [34]. The higher metabolic rate of in vitro produced embryos is evidenced in our in vitro hatched blastocysts by an enrichment in tRNA charging and amino acid metabolism. This transcriptional profile is in line with the proposed quiet embryo hypothesis, where viability is highest for embryos with a low rate of metabolism [37]. In addition, embryos with high DNA damage display an increased amino acid turnover [38, 39]. Thus, we propose that the transcriptome of in vitro produced hatched blastocysts is indicative of an increased level of DNA damage, as evidenced by the higher degree of amino acid metabolism. The effect of adding higher concentrations of arginine, i.e., 0.36 mM instead of 0.1 mM, to the embryo culture medium on the embryos’ amino acid metabolism and DNA damage should be evaluated. Thereby, an improvement of the currently employed in vitro fertilization pipelines can be assessed.

## Conclusions

Taken together, we show that early developing in vivo and in vitro produced embryos display largely similar transcriptome profiles. Embryos with compromised developmental competence are likely arrested at an early stage of development. At the blastocyst stage, only few differences persisted between in vivo and in vitro, and there was no transcriptional difference between male and female embryos. The in vitro produced hatched blastocysts displayed the  expression of transcripts indicative of a higher metabolic rate and the arginine transporter, suggesting a lower developmental competence compared to the in vivo developed embryos.

## Methods

### Embryo production

Porcine embryos were allowed to develop in vivo and were produced in vitro (Fig. 1). The development-specific transcriptome dynamics were investigated by analyzing 4-cell stage embryos, morulae and hatched blastocysts. At the hatched blastocyst stage, male and female in vivo embryos were compared to the respective in vitro produced embryos.

### In vivo

The in vivo embryos were produced as described previously [16]. In brief, 12 German Landrace × Pietrian crossbred gilts were kept at the Research station Thalhausen of the Technical University of Munich, Germany. The gilts were synchronized using Altrenogest ReguMate® for 12 days. Intergonan® (PMSG) was applied once on the following evening at 750 iU. Ovogest® (human chorion gonadotropin) was applied 3.5 days later at 750 iU. The next day (day 0), all animals were inseminated with sperm of the same Duroc boar, named SWIROC. On day 2, 4 and 6 post insemination, four gilts were randomly selected, stunned by electro-anesthesia and slaughtered by bleeding in a commercial slaughterhouse to retrieve the embryos. The reproductive tracts were collected immediately after slaughter and the embryos were recovered from the reproductive tracts by flushing. The day 2 embryos were flushed from the oviduct with 2 ml phosphate buffered saline (PBS), while on day 4 and 6, embryos were flushed from the uterus with 10 ml PBS per horn. The collected embryos were washed twice with fresh PBS and single embryos were transferred to a cryotube and snap frozen in liquid nitrogen. All samples were stored at − 80 °C until library preparation. At 2, 4 and 6 days after insemination, 4-cell embryos, morulae and hatched blastocysts were collected. Per group, *n* = 5–10 embryos were randomly selected, stemming from three to four gilts.

### In vitro

The in vitro embryos were produced as previously described [40, 41]. In brief, antral follicles on the surface of ovaries obtained from a local abattoir with a size of 3–6 mm in diameter were aspirated for the collection of cumulus-oocyte complexes (COCs) [41]. The maturation of COCs displaying more than three layers of compact cumulus cells took place by culturing them in FLI medium contained FGF2, LIF and IGF1 for 44–46 h [36]. During the first 22 h, the COCs were cultured in maturation medium supplemented with human chorionic gonadotropin and pregnant mare serum gonadotropin, followed by 22–24 h of culture in hormone free maturation medium in a humidified atmosphere of 5% CO_2_, 5% O_2_ and 90% N_2_ at 38.5 °C [41]. The in vitro fertilization was performed using frozen sperm derived from the same Duroc boar as used for the in vivo developed embryos to reduce an influence on genetic variation [40]. A group of 20 matured oocytes was co-incubated for 7 h with 1.0 × 10^6^ cells/mL in a porcine fertilization medium (Functional PeptideCo., Yamagata, Japan) in a humidified atmosphere of 5% CO_2_, 5% O_2_ and 90% N_2_ at 38.5 °C [40]. After fertilization, the cumulus cells and excess sperm were removed from the presumed zygotes and were cultured in Porcine Zygote medium-5 (Functional Peptide Co., Yamagata, Japan) in a humidified atmosphere of 5% CO_2_, 5% O_2_, and 90% N_2_ at 38.5 °C [40]. The embryos were produced in four independent experiments. Morphologically normal embryos of 4-cell stage, compacted morulae and hatched blastocysts were collected at the following time points after fertilization, respectively: 48 h, 100 h and 174 h. 4-cell stage embryos and compacted morulae were especially collected from a population of preselected 2-cell embryos at 30 h after fertilization to avoid sampling of abnormal embryos. Prior to freezing, the embryos were washed trice with PBS containing 0.1% PVA. The embryos were transferred to a 0.5 ml Eppendorf tube and snap frozen in liquid nitrogen. Samples were stored at − 80 °C until library preparation. Per group, *n* = 5–10 embryos were randomly selected, stemming from three to four experiments.

### RNA sequencing

Single 4-cell stage embryos (n = 5/production method), morulae (*n* = 10/production method) and hatched blastocysts (n = 10/production method) were obtained from in vivo flushing or were in vitro produced (Fig. 1). The library preparation for RNA-sequencing was conducted as previously described [42]. A total of 18 PCR cycles was used for the library preparation. Single embryos were lysed in 1 μl lysis buffer containing dNTPs and tailed oligo-dT oligonucleotides (30 nt poly-dT stretch and 25 nt universal 5’anchor sequence) plus 3.1 μl PBS [42]. The lysed embryos were subjected to cDNA synthesis and library preparation with the Smart-seq2 protocol as described previously [42]. The libraries were pooled and sequenced on the NovaSeq6000 with a sequencing depth of 14 ± 4 million reads per sample (mean ± SD).

### Data analyses and bioinformatics

Raw sequence reads (Fastq files) were analyzed on a locally installed Galaxy system [43]. Basic read statistics and read quality was evaluated based on FastQC reports [44], and a MultiQC overview report of all samples was generated [45]. Adaptors were clipped, sequences shorter than 20 bp were removed, and a low-quality end score of 20 was applied with the Trim Galore! tool [46]. The trimmed reads were aligned against the porcine genome (*Sus scrofa* 11.1) with HISAT2 [47]. The mapping rate was 84 ± 6% (mean ± SD). An additional sequencing quality control was included. The reads of three representative and most abundant transcripts were aligned and visualized with the Integrative Genomics Viewer (IGV, version 2.8.2). The sex of both morulae and hatched blastocysts was assigned based on the expression of *DDX3Y*, *EIF1AY* and *EIF2S3Y* [16, 48]. Even though the morulae still had sperm attached to their zona, females were identified based on the absence of expression of the Y-chromosome specific genes. The 4-cell embryos were not sexed, as they were sampled around the time of EGA and as sperm were still attached to the zona. A between group analysis was conducted in R (version 3.6.1) [49]. A self-organizing tree algorithm was ran for both embryo production methods to visualize the developmental dynamics [50]. Differential gene expression analyses was conducted with EdgeR [51]. A false discovery rate (FDR) of < 0.1% and an absolute log_2_FC > 1 was applied to identify the differentially expressed genes (DEGs), which had a CPM > 0.5 in at least one of the replicates per experimental condition. The identified DEGs were used for pathway enrichment analyses [52]. The functional analysis was conducted with the Qiagen Ingenuity Pathway Analysis (IPA) software. Human orthologues of DEGs were identified with the Mammalian Annotation Database for improved annotation and functional classification of Omics datasets from less well-annotated organisms [53]. A total of 21,211 21211 porcine genes were expressed and 17,219 17219 human orthologues were identified. To conduct canonical pathway analyses, different log_2_ FC cut-offs were set to prevent an enrichment of redundant and overly general pathways [52]. To prevent overly general pathway enrichments, a maximum of 3000 DEGs should be used, while allowing the inclusion of as many DEGs as possible (Qiagen IPA user manual). In addition, to prevent functional enrichment analysis biases, the background “universe” (Additional file 4) was defined by all genes detected in the RNA-seq experiment [54]. An absolute log_2_ FC cut-off of 6 was applied to obtain 3063 DEGs for canonical pathway analysis for the in vivo 4-cell to morula stage, while a log_2_ FC cut-off of 1.7 was applied to obtain 1559 DEGs for canonical pathway analysis for the in vivo morula to hatched blastocyst stage. An absolute log_2_ FC cut-off of 4 was applied to obtain 2616 DEGs for canonical pathway analysis for the in vitro 4-cell to morula stage, while a log_2_ FC cut-off of 1.7 was applied to obtain 2656 DEGs for canonical pathway analysis for the in vitro morula to hatched blastocyst stage. An absolute log_2_ FC cut-off of 0.8 was applied to obtain 377 DEGs for canonical pathway analysis for the in vivo versus in vitro hatched blastocysts. Canonical pathways were considered statistically significant with a *p* ≤ 0.05 and an absolute z-score > 2.

## Supplementary Information

**Additional file 1. **Embryonically detected transcripts. The detected transcripts for each developmental stage and production method are displayed as violin plot, boxplot and individual data points. Letters in the graph indicate statistically significant differences (*p* < 0.05) between the developmental stages for each production method.

**Additional file 2.** cDNA profiles and library smear analysis of in vivo developed and in vitro produced 4-cell embryos, morulae and hatched blastocysts.

**Additional file 3.** Read alignments and coverage of *SDHD*, *DNMT1*, and *KPNA7* for the 4-cell embryos, *SDHD*, *DNMT1*, and *UEBE2* for the morulae, and *DNMT1*, *FTH1*, and *FABP3* for the hatched blastocysts.

**Additional file 4.** Background “universe” used for Ingenuity Pathway Analysis which includes all identified genes in the RNA-seq experiment and the human orthologues used for the biological function analyses with the© 2000–2020 QIAGEN Ingenuity Pathway Analysis software.

## Data Availability

The dataset generated and analyzed during the current study are available in the National Center for Biotechnology Information (NCBI) Gene Expression Omnibus, https://www.ncbi.nlm.nih.gov/geo/query/acc.cgi?acc= GSE155043.

## Abbreviations

COCs: cumulus-oocyte complexes
DEGs: differentially expressed genes
EGA: embryonic genome activation
E2: estradiol-17β
ICM: inner cell mass
IPMK: inositol polyphosphate multikinase
PCA: principal component analyses
RAC1: Rac family small GTPase 1
SCNT: somatic cell nuclear transfer
TE: trophectoderm
