# Supplementary material for: Transcriptome dynamics in early in vivo developing and in vitro produced porcine embryos
Source: BMC Genomics. 2021 Feb 27;22:139. doi: 10.1186/s12864-021-07430-7 (PMC7913449; doi:10.1186/s12864-021-07430-7)
Supplement: Supplementary file 1 — Additional file 1. Embryonically detected transcripts. The detected transcripts for each developmental stage and production method are displayed as violin plot, boxplot and individual data points. Letters in the graph indicate statistically significant differences (p < 0.05) between the developmental stages for each production method. [file 12864_2021_7430_MOESM1_ESM.pdf]

Additional file 1: Embryonically detected transcripts

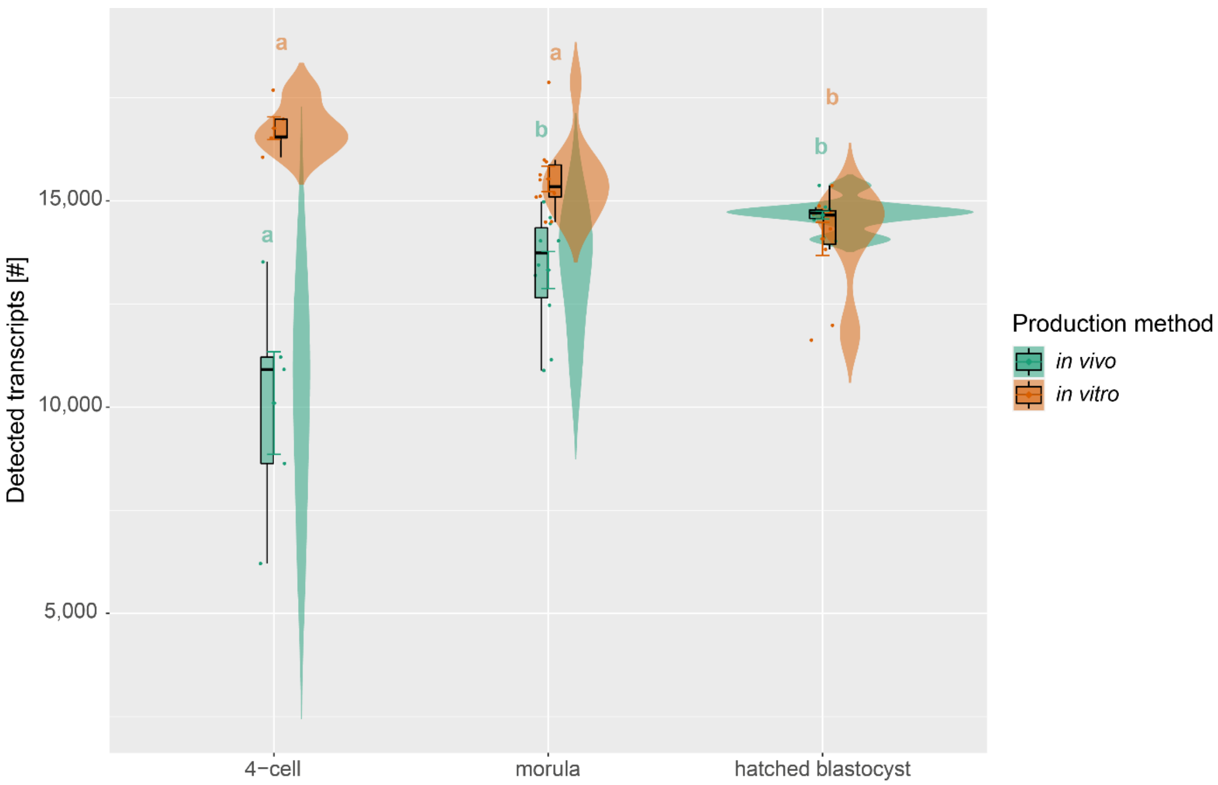

Additional file 1. Embryonically detected transcripts. The detected transcripts for each developmental stage and production method are displayed as violin plot, boxplot and individual data points. Letters in the graph indicate statistically significant differences (p<0.05) between the developmental stages for each production method.
