# Supplementary material for: Transcriptome dynamics in early in vivo developing and in vitro produced porcine embryos
Source: BMC Genomics. 2021 Feb 27;22:139. doi: 10.1186/s12864-021-07430-7 (PMC7913449; doi:10.1186/s12864-021-07430-7)

Additional file 2: cDNA profiles and library smear analysis

Porcine embryos cDNA profiles

In vivo 4-cell embryo

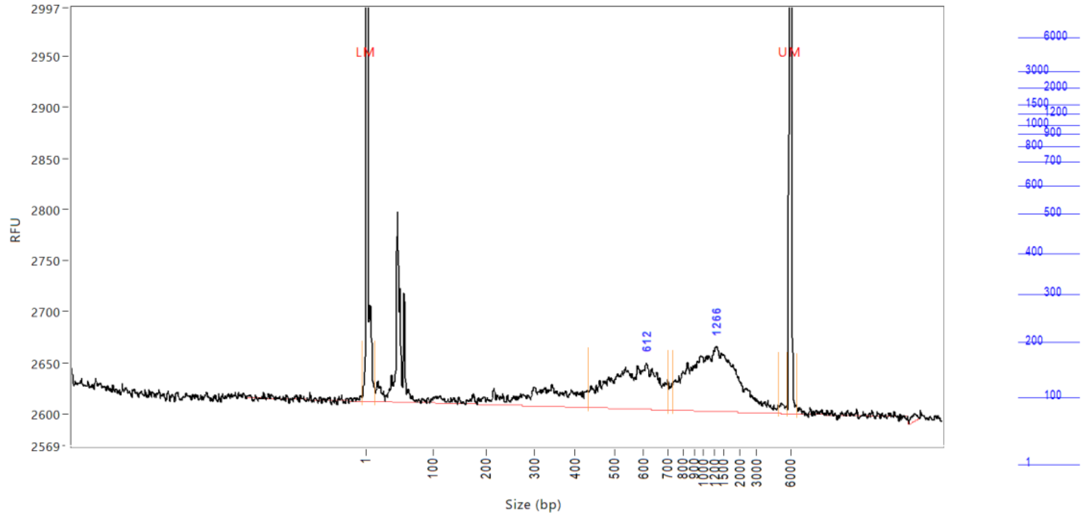

In vitro 4-cell embryo

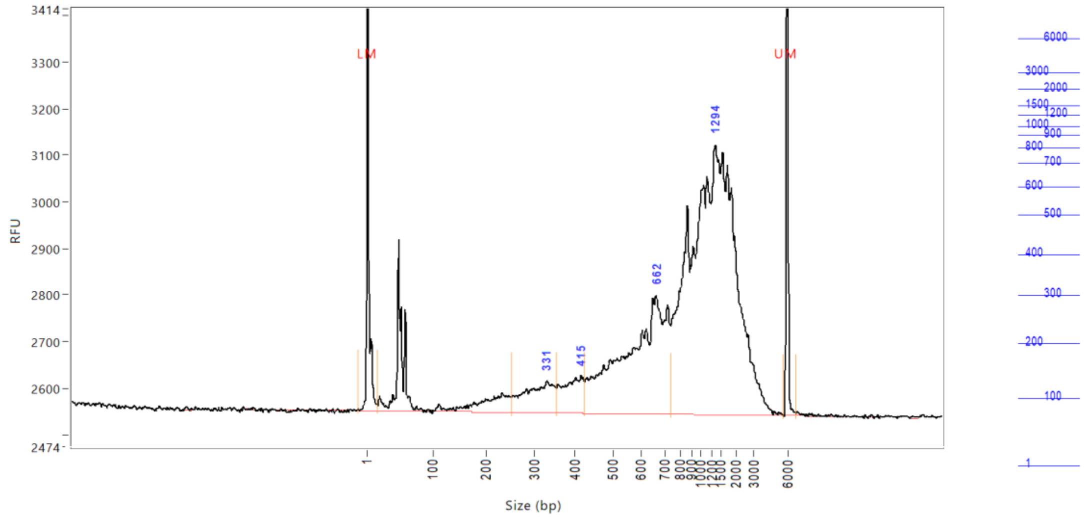

Additional file 2: cDNA profiles and library smear analysis

In vivo morula

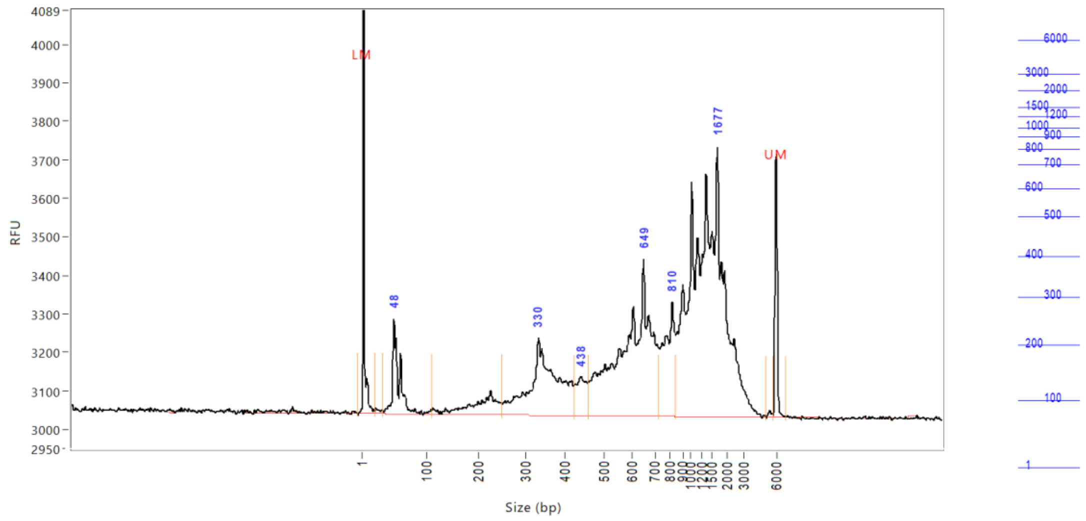

In vitro morula

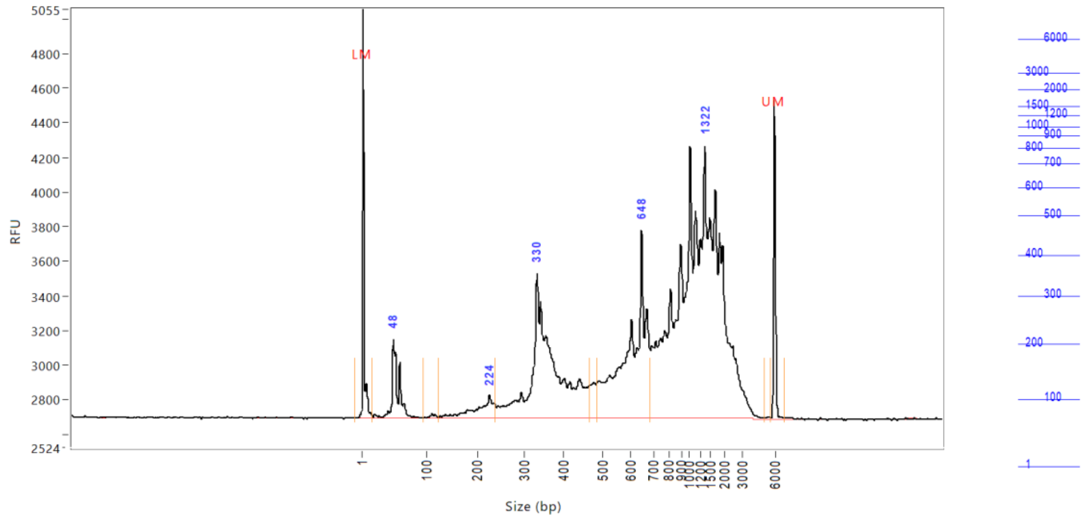

Additional file 2: cDNA profiles and library smear analysis

In vivo hatched blastocyst

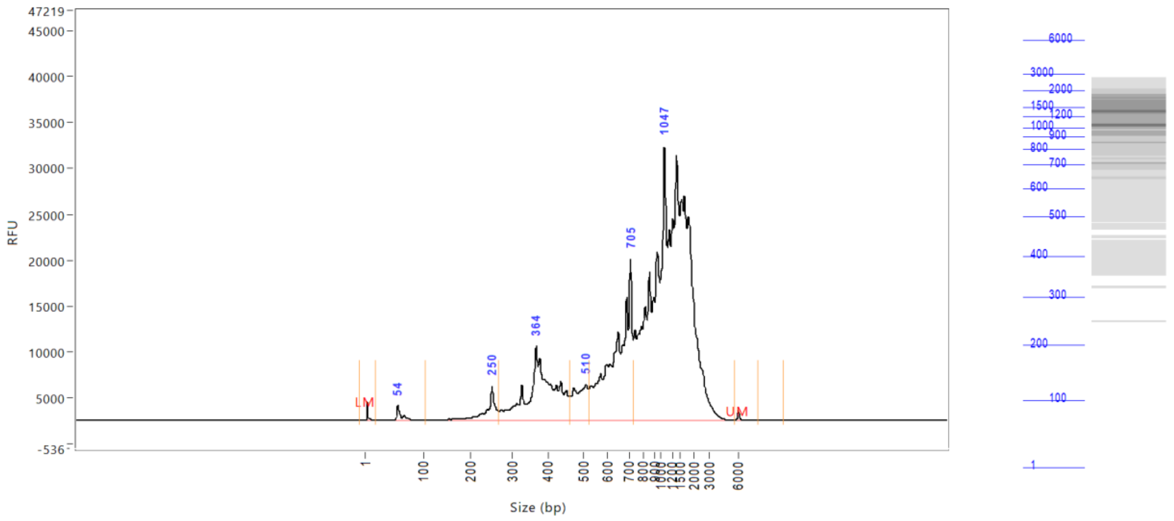

In vitro hatched blastocyst

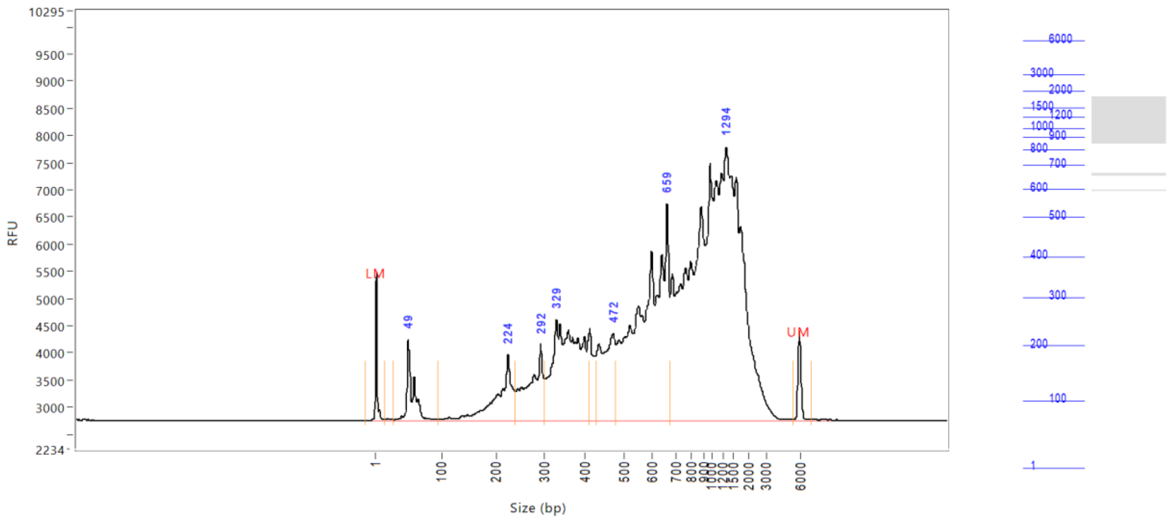

Additional file 2: cDNA profiles and library smear analysis

Porcine embryos library smear analysis

In vivo 4-cell embryo

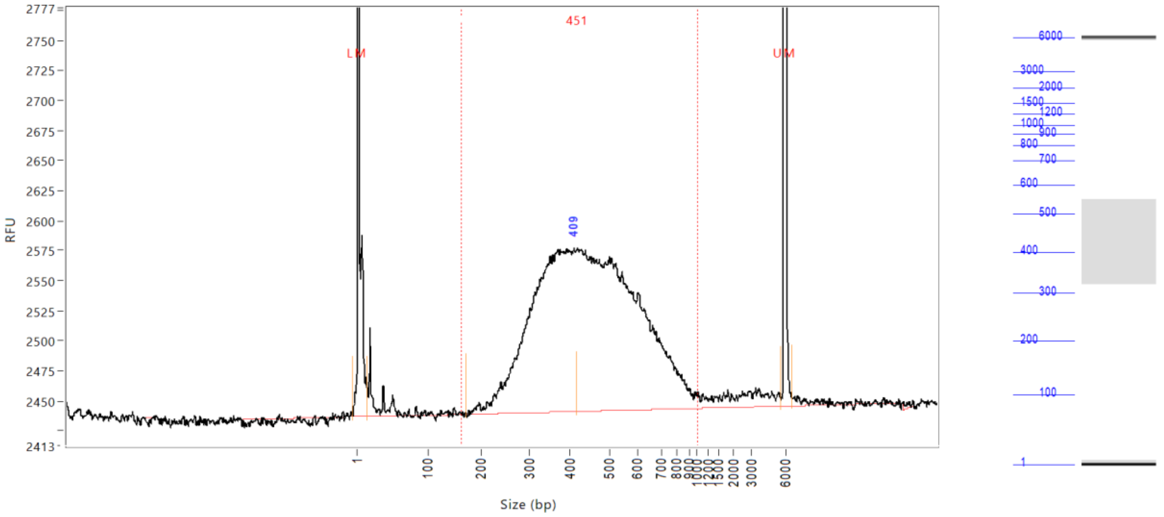

In vitro 4-cell embryo

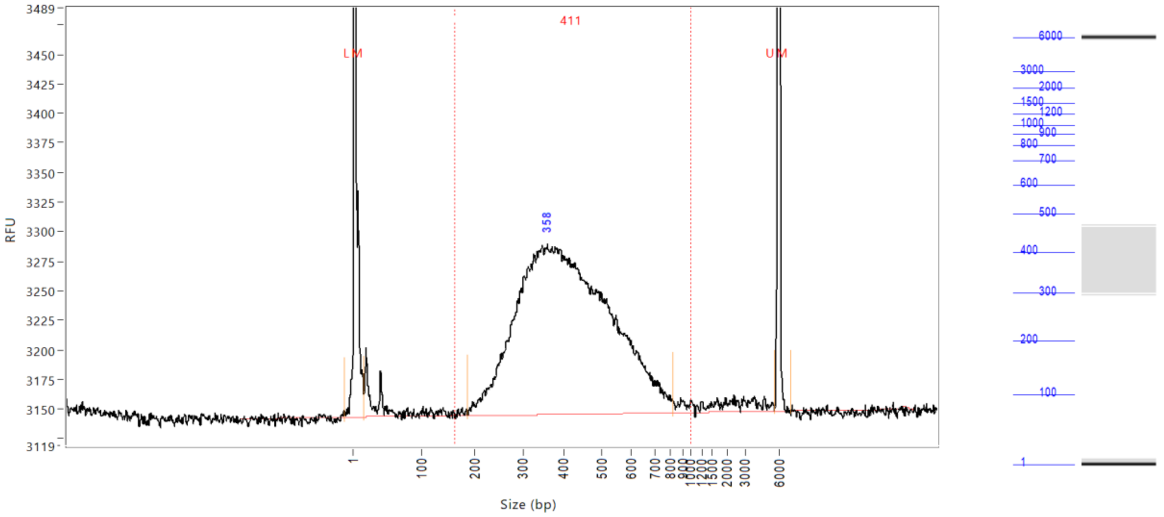

Additional file 2: cDNA profiles and library smear analysis

In vivo morula

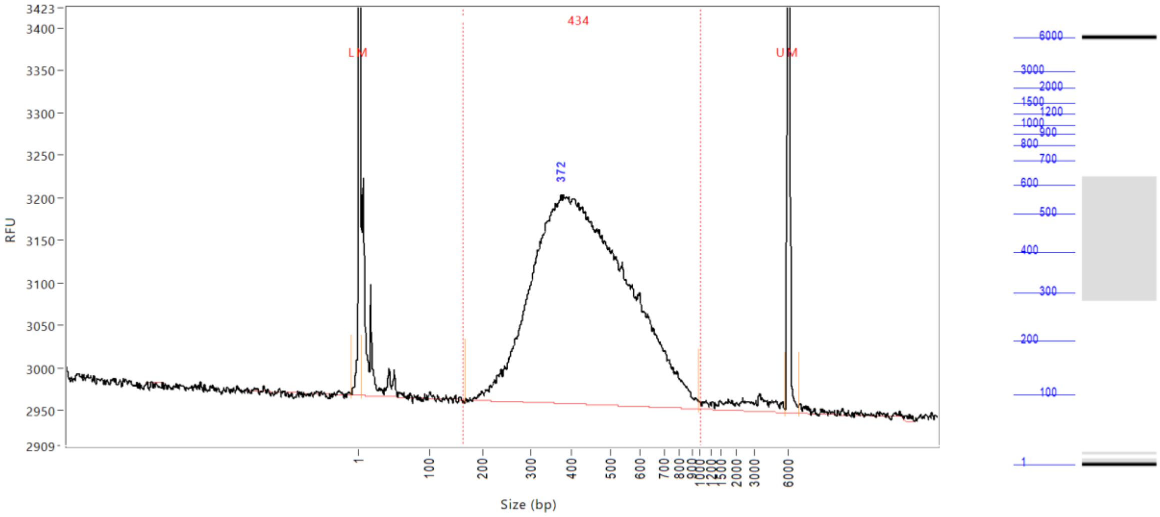

In vitro morula

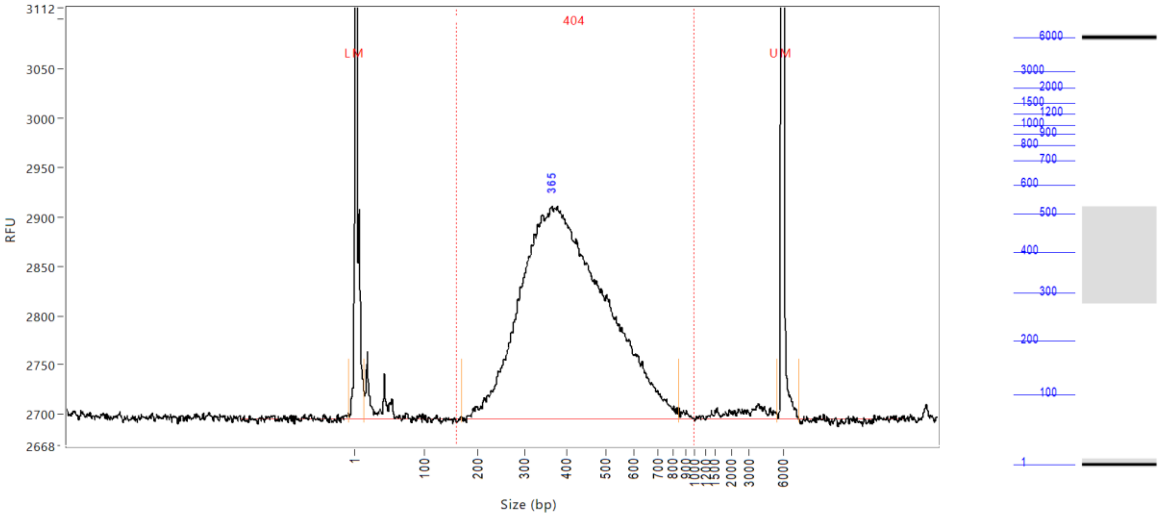

Additional file 2: cDNA profiles and library smear analysis

In vivo hatched blastocyst

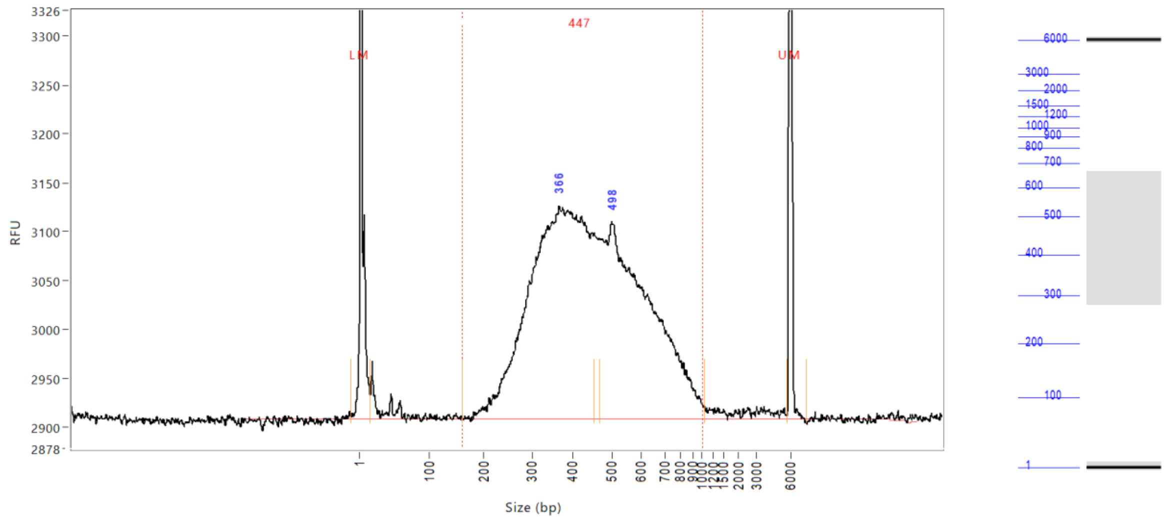

In vitro hatched blastocyst

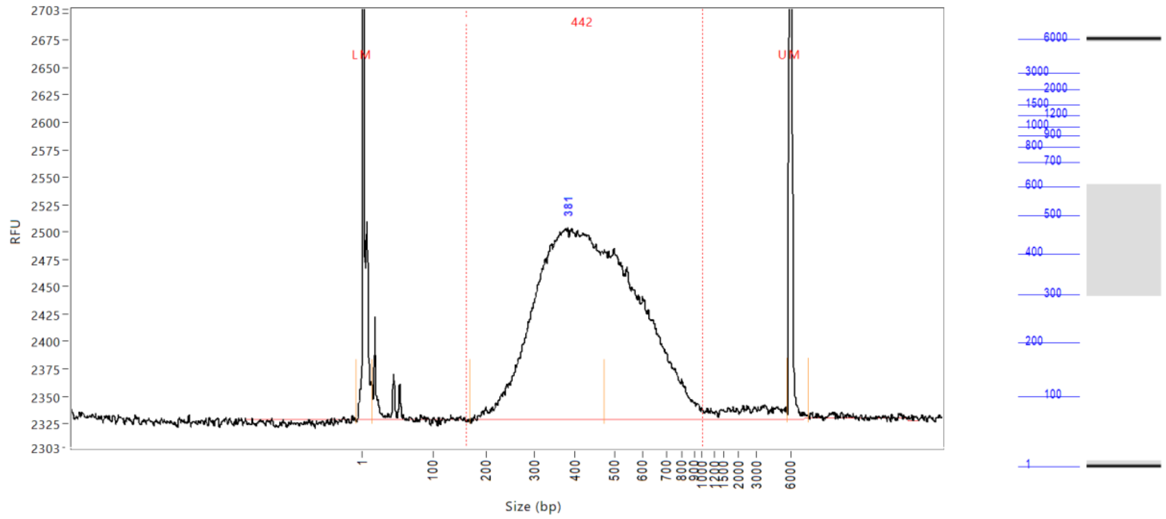

Supplement: Supplementary file 2 — Additional file 2. cDNA profiles and library smear analysis of in vivo developed and in vitro produced 4-cell embryos, morulae and hatched blastocysts. [file 12864_2021_7430_MOESM2_ESM.pdf]
