# Supplementary material for: Transcriptome dynamics in early in vivo developing and in vitro produced porcine embryos
Source: BMC Genomics. 2021 Feb 27;22:139. doi: 10.1186/s12864-021-07430-7 (PMC7913449; doi:10.1186/s12864-021-07430-7)

Additional file 3: read alignment coverage

Alignment coverage 4-cell embryos

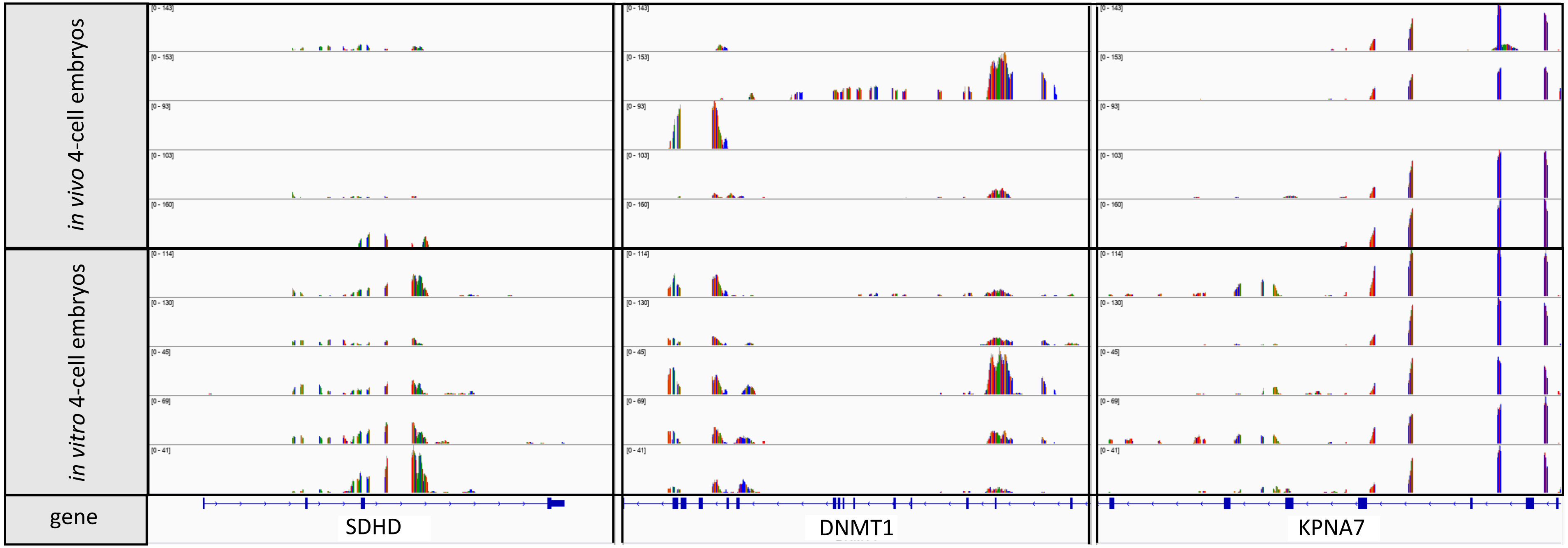

Additional file 3: read alignment coverage

Alignment coverage morulae

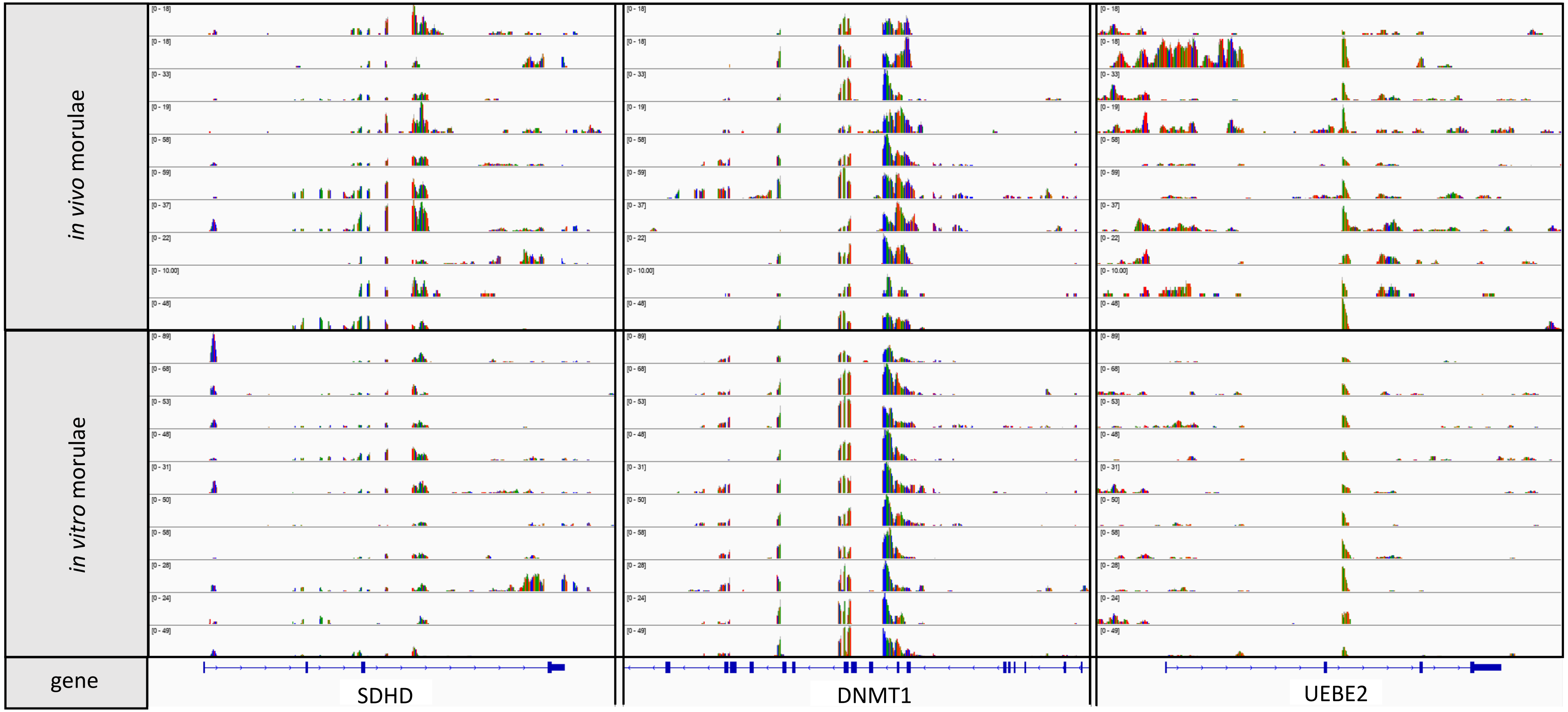

Additional file 3: read alignment coverage

Alignment coverage hatched blastocysts

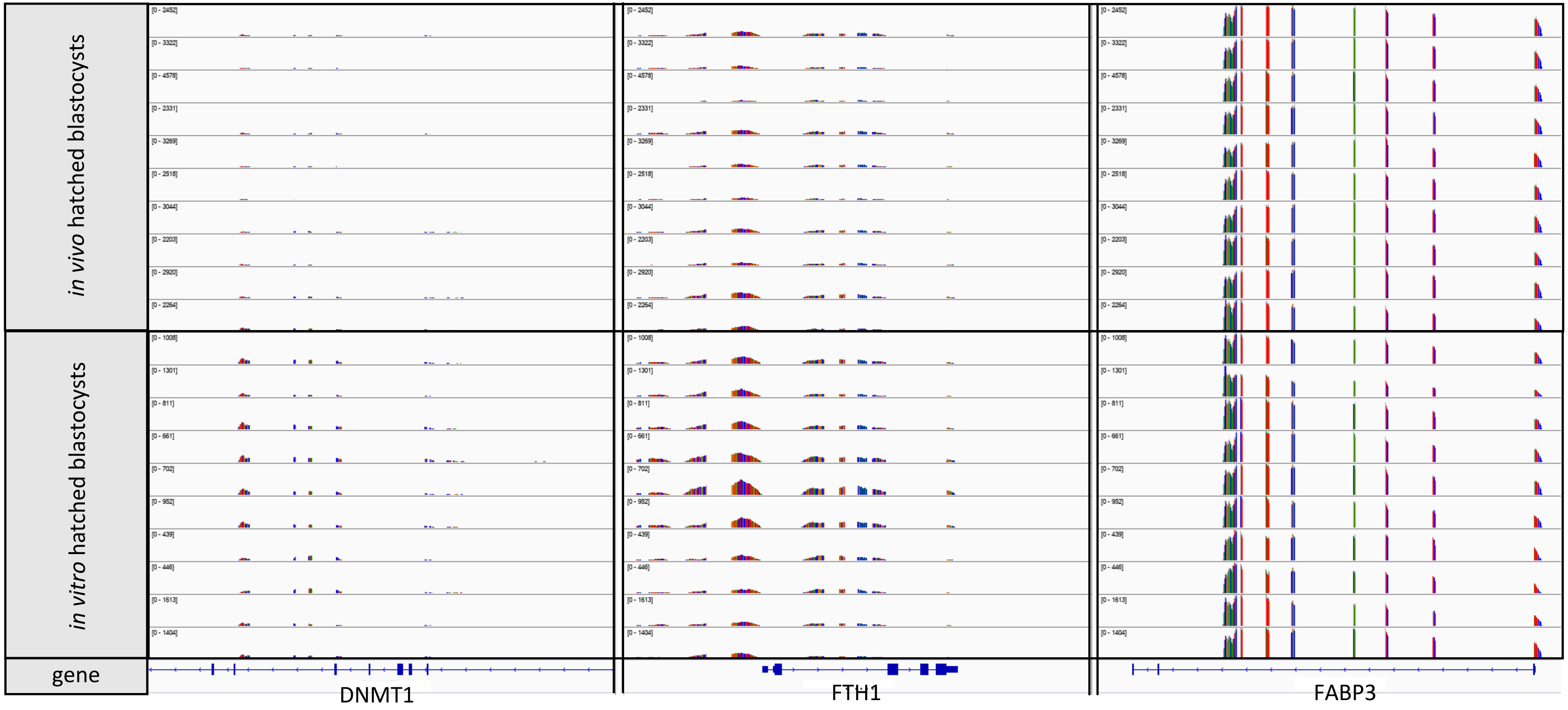

Supplement: Supplementary file 3 — Additional file 3. Read alignments and coverage of SDHD, DNMT1, and KPNA7 for the 4-cell embryos, SDHD, DNMT1, and UEBE2 for the morulae, and DNMT1, FTH1, and FABP3 for the hatched blastocysts. [file 12864_2021_7430_MOESM3_ESM.pdf]
